# IKKα inhibition re-sensitizes acquired adriamycin-resistant triple negative breast cancer cells to chemotherapy-induced apoptosis

**DOI:** 10.1038/s41598-023-33358-x

**Published:** 2023-04-17

**Authors:** Jian Liao, Qing-hong Qin, Fa-you Lv, Zhen Huang, Bin Lian, Chang-yuan Wei, Qin-guo Mo, Qi-xing Tan

**Affiliations:** 1 Department of Breast Surgery, Guangxi Medical University Cancer Hospital, 71 Hedi Road, Nanning, 530021 Guangxi Province People’s Republic of China; 2 Key Laboratory of Breast Cancer Diagnosis and Treatment Research of Guangxi, Department of Education, Nanning, 530021 Guangxi Province People’s Republic of China

**Keywords:** Cancer, Chemical biology, Genetics, Diseases

## Abstract

IKKα has been shown to be responsible of multiple pro-tumorigenic functions and therapy resistance independent of canonical NF-κB, but its role in acquired chemotherapy resistance in breast cancer remains unclarified. In this study, we obtained pre-treatment biopsy and post-treatment mastectomy specimens from a retrospective cohort of triple-negative breast cancer (TNBC) patients treated with neoadjuvant chemotherapy(NAC) (n = 43). Immunohistochemical methods were used to detect the expression of IKKα before and after NAC, and the relationship between IKKα and the pathologic response to NAC was examined. In addition, we developed a new ADR-resistant MDA-MB-231 cell line(MDA-MB-231/ADR) and analyzed these cells for changes in IKKα expression, the role and mechanisms of the increased IKKα in promoting drug resistance were determined in vitro and in vivo. We demonstrated that the expression of IKKα in residual TNBC tissues after chemotherapy was significantly higher than that before chemotherapy, and was positively correlated with lower pathological reaction. IKKα expression was significantly higher in ADR-resistant TNBC cells than in ADR-sensitive cells, IKKα knockdown results in apoptotic cell death of chemoresistant cells upon drug treatment. Moreover, IKKα knockdown promotes chemotherapeutic drug-induced tumor cell death in an transplanted tumor mouse model. Functionally, we demonstrated that IKKα knockdown significantly upregulated the expression of cleaved caspase 3 and Bax and inhibited the expression of Bcl-2 upon ADR treatment. Our findings highlighted that IKKα exerts an important and previously unknown role in promoting chemoresistance in TNBC, combining IKKα inhibition with chemotherapy may be an effective strategy to improve treatment outcome in chemoresistant TNBC patients.

## Introduction

Breast cancer is the most prevalent cancer in women worldwide. Triple-negative breast cancer (TNBC) is an aggressive subtype of breast cancer, and it is characterized by the absence of Estrogen receptor (ER), Progesterone receptor (PR) and Human epidermal growth factor receptor-2 (HER-2) on the surface of tumor cell membrane. TNBC accounts for 15 to 20% of breast cancer cases and 25% of breast cancer-associated deaths. TNBC is more likely to metastasize and relapse at early stages than other breast cancer subtypes and is associated with shorter overall survival (OS)^1^. In addition, due to the lack of corresponding therapeutic targets, TNBC can not benefit from endocrine therapy or targeted therapy, chemotherapy is currently the primary systemic treatment for TNBC. Adriamycin is a cytotoxic drug that inhibits RNA and DNA synthesis and is widely used in the treatment of TNBC due to its high efficacy. Unfortunately, the development of chemoresistance remains a major hurdle to its use and limits its effectiveness^2^. Accordingly, it is crucial to understand the mechanisms that drive the resistance of TNBC cancer cells to adriamycin, in order to develop strategies that can limit resistance and improve the outcomes of TNBC patients.

Chemotherapy drugs often activate the cellular stress response of tumor cells, and these stress responses may further lead tumor cells to activate their own protective and defense mechanisms, and thereby increase the resistance of tumor cells to chemotherapy drugs. The IKK/ nuclear factor-kappa B (NF-κB) pathway is a key signaling pathway mediating cell stress response, plays a crucial role in carcinogenicity, tumor cell proliferation, and chemotherapy resistance in several malignancies^3^. This pathway mainly comprises the transcription factor NF-κB, repressor I-κB and protein kinase IKK. The protein kinase IKK complex comprises catalytic subunits IKKα, IKKβ, and a regulatory subunit IKKγ. Ikkα and IKKβ catalyze IκBα by exerting kinase activity and then phosphorylates IκBα. The IκBα substrate molecules undergo an inducible phosphorylation modification, which promotes the latter to be degraded by poly-ubiquitination and subsequent proteasomal degradation. This allows the release and transportation of NF-κB into the nucleus to regulate the transcription of downstream related target genes^4^. In fact, the function of the IKK kinase complex is not limited to the well-known NF-κB signaling pathway. Studies have shown that IKKα can enhance the metastatic activity of prostate and squamous cell carcinoma by regulating the Maspin gene^5,6^. Animal studies have shown that IKKα activation is required for the occurrence and progression of bowel cancer and lung adenocarcinoma^7,8^. During nutrient starvation, IKKα and IKKβ are involved in inducing autophagy by up-regulating the expression of essential autophagy genes^9^. A recent study has shown that IKKα kinase mediates chemotherapy resistance in tumor cells by regulating DNA damage response, and this effect is independent of the induced activation state of NF-κB^10^. More and more new functions of IKK kinase unrelated to NF-κB have been discovered, which are mainly involved in tumorigenesis, stress response, transcriptional regulation, immune function and cell cycle regulation, suggesting that IKK kinase can play a variety of biological functions in an independent way of NF-κB. Recently, we found that IKKα can regulate p53-dependent autophagy independently of NF-κB and play a protective role against apoptosis during arsenice-induced chemical toxicity^11^. Given that IKKα mediates apoptotic response induced by cytotoxic chemical stimulators, we explored whether it is central to the apoptotic effects induced by other chemotherapies. Adriamycin is the most commonly used cytotoxic drug in breast cancer chemotherapy, we previously reported that adriamycin stimulation can cause cellular stress response, this may be an important mechanism of drug resistance in triple negative breast cancer^12^. However, whether IKKα participates in adriamycin-induced resistance remains to be validated.

In the present study, we have assessed the role of IKKα in chemotherapy resistance using clinical samples and an experimental cell-culture model. We detected IKKα expression in residual triple-negative breast cancer tissues after NAC and compared it with that before NAC. We also used a pulse stimulated strategy to mimic the clinical effects of chemotherapy to generate triple negative MDA-MB-231 cells that are resistant to ADR relative to parental cells (MDA-MB-231/ADR), and the role and mechanisms of IKKα in promoting ADR resistance were determined in vitro and in vivo. We showed that the increased IKKα contributed to ADR-induced resistance and that genetic inhibition of IKKα re-sensitized the resistant MDA-MB-231 cells to ADR. Our study can help us better understand the molecular and biological involvement of IKKα in TNBC and drug resistance.

## Material and methods

### Immunohistochemistry assay

Primary tissues were obtained from TNBC patients.who did not achieve a complete pathological response to neoadjuvant chemotherapy. The pathological response of chemotherapy was evaluated according to the Miller-Payne (MP) classification system^13^. Immunostaining was performed using the MaxVision kit (Maixin Biol, Fuzhou, China) following the manufacturer's instructions. Samples were incubated overnight at 4 °C with primary antibodies against IKKα (1:50, CST#61294), rinsed with PBS and incubated with horseradish peroxidase-conjugated secondary antibody for 20 min at room temperature (MaxVision™ two-step system:KIT-5010; Maixin Biotechnology Co. Ltd). The tissues were finally incubated with diaminobenzidine for 3 min. Tissue FAXS Viewer system was used for imaging and analysis of 3 independent and randomly selected sections within the tumor area of each slide. The expression of IKKα in tissues was assessed by individuals blinded to the experiment using the immunoreactive score (IRS). IKKα was quantified based on staining intensity and percentage of stained tumor cells. The staining proportion were classified into five categories as follows: 0 for 0% stained, 1 for < 25% stained, 2 for 25–50% stained, 3 for 50–75% stained, and 4 for 75–100% stained. The staining intensity was classified from 0 to 3: 0 (−), 1 (+), 2 (++), or 3 (+++). The final IKKα results were obtained by multiplying the extent of staining with the intensity scores, resulting in an IRS score between 0 and 12. A cut-off for high IKKα expression was set at IRS ≥ 6.The pathological sections for immunohistochemistry were excised specimens obtained for pathological testing during surgery at Guangxi Medical University Cancer Hospital. The study followed the Declaration of Helsinki and was approved by the ethics department of the Cancer Affiliated Hospital of Guangxi Medical University. Immunohistochemical staining analysis was performed on anonymous tissue specimens. Therefore, the need for the patient's written informed consent was waived by The Medical Ethics Committee of Guangxi Medical University Cancer Hospital. The study was approved by the Ethical Review Committee of Guangxi Medical University Cancer Hospital(Approval Number: LW2023015).

### Cell culture

TNBC cell lines MDA-MB-231 and MDA-MB-231/ADR were purchased from the Cell Bank of Type Culture Collection of the Chinese Academy of Sciences (Shanghai Yansheng Industrial Co., LTD, Shanghai, China). Adriamycin-resistant MDA-MB-231/ADR cells were screened from MDA-MB-231 cells exposed to adriamycin in different doses under optimal growth conditions (10 ng/mL of adriamycin in the beginning, and gradually increasing the dose to 2ug/mL), the survival of adriamycin-resistant cell line MDA-MB-231/ADR cells after half a year of screening). MDA-MB-231 cells were cultured in DMEM, while MDA-MB-231/ADR cells were cultured in L15 medium supplemented with 10% FBS and 1% Penicillin–streptomycin. The cells were incubated at 37 °C in 5% CO_2_ environment. MDA-MB-231/ADR cells were specifically cultured in a medium supplemented with adriamycin (500 ng/ml) to sustain the drug-resistant phenotype.All cells were cultured in a drug-free medium for 36 h before in vitro assay.We regularly used mycoplasma reagents to monitor mycoplasma, and there was no mycoplasma contamination.

### Transfection with lentivirus

During transfection with lentivirus, the shRNAs against IKKα included shRNA#1 (5'-gcAAATGAGGAACAGGGCAAT-3'), shRNA#2 (5'-gcGTGCCATTGATCTATATAA-3'), and shRNA#3 (5'-ccAGCCTCTCAATGTGTTCTA-3'). lv-RNA used against IKKα was lv-RNA (F primer: AGGTCGACTCTAGAGGATCCCGCCACCATGGAGCGGCCCCCGGGGCTGCG, primer R primer: TCCTTGTAGTCCATACCTTCTGTTAACCAACTCCAATCAAGATTC). The cells were then transfected with IKKα shRNAs, control shRNA and IKKα lvRNA, control lvRNA (Genechem Shanghai, China) for 72 h. Puromycin (5 μg/mL) was used to screen for puromycin-resistant cell populations.

### Viability assays

Cell number was monitored with CCK-8(Meilunbio,Dalian, China) according to the manufacturer’s instructions. The doses corresponding to the IC50 values were calculated with SoftMax software.

### Apoptosis analysis

Harvested cells were washed with cold PBS and centrifuged at 2000 rpm for 5 min. The cells (1 × 10^6^) were resuspended in 500 μl Binding Buffer, incubated with 5 μl of AnnexinV-APC and 10 μl of 7-AAD (MultiSciences LiankeBio, China) for 15 min at room temperature under the light before flow cytometry (FACS Calibur, BD Biosciences) analysis for cell apoptosis rate and at least 10,000 events were recorded.The analysis of FSC files was by Flow Jo (v10.8.1).

### Western blotting assay

Cells were processed in RIPA buffer (Beyotime, China) at 4 °C for 30 min. The proteins were separated using SDS-PAGE and then proteins were transferred to Polyvinylidene Fluoride(PVDF) membranes. The membrane is cut to size according to the location of the gene protein.and then the nitrocellulose membranes were blocked with 5% BSA (Beyotime, China) for 2 h at room temperature on a rocker. The membranes were then incubated overnight at 4 °C with the primary antibodies (1:1000 dilution), including anti IKKα (CST#61294), p-IKKα (CST#2697S), Bax (Bioss, #bs-0127R), Bcl-2 (Bioss, #bs-0032R), caspase3 (Bioss, #bs-20364R), and GAPDH (CST #5174). Then, incubated with HRP-conjugated anti-IgG secondary antibodies (Zhongshanjinqiao, China) at 1:2500 dilution for 2 h at room temperature on a rocker. The membranes were scanned using an enhanced chemiluminescence detection system (Vision Works).

### Quantitative real time-PCR (RT-PCR)

Total RNA was extracted using TRIzol (Ambion, USA), according to the manufacturer's protocol. RNA (1 μg) was reverse transcribed into cDNA using the Prime ript RT Master Mix (Takala, Dalian, China). qPCR was performed using the qTOWER3 platform (Analytik Jena AG, Germany) using SYBR PreMix ex Taq benchmark (Takala, Dalian, China)^14^. The Sequences of primers used for qPCR were as follows: 5'-ATGAAGAAGTTGAACCATGCCA-3' and 5'-CCTCCAGAACAGTATTCCATTGC-3' were F and R primers for IKKα。5'-GCACCGTCAAGGCTGAGAAC-3' and 5'-TGGTGAAGACGCCAGTGGA-3' were F and R primers for GAPDH-R. The amplification was calculated using the 2^−ΔΔCq^method^15^.

### In vivo experiments

Female 4-weeks old athymic BALB/c nude mice were purchased from the experimental animal center of Guangxi Medical University (Guangxi, China). Briefly, 3 × 10^6^ MDA-MB-231/ADR cells (con-shRNA,shIKKα# 2, shIKKα# 3) were diluted with 0.1 mL PBS containing 50% Matrigel(Corning, NY, USA) and then injected subcutaneously into the mammary fat pad or axilla of the mice. Treatments started when the tumor reached an average volume 50–55 mm^3^. Mice were treated with saline, adriamycin (1.5 mg/kg) or both.

By oral gavage every 3 days. The tumor volumes and body weights of mice were measured every 3 days. The tumor masses were extracted after 21 days. At the end of the experiment, mice were sacrificed; tumor xenografts were isolated, weighed, and fixed in formalin for hematoxylin & eosin (H & E) staining. The expression of IKKα protein and apoptosis-related proteins were evaluated using Immunohistochemical (IHC) analysis. Animal experiments and procedures comply with the ARRIVE guidelines and were approved by the Animal Care and Use Committee of Guangxi Medical University and performed in accordance with the Guide for the Care and Use of Laboratory Animals (The Tab of Animal Experimental Ethical Inspection No: 202012021). Our animal experiments were performed in accordance with relevant guidelines and regulations.

### Statistical analysis

All statistical analyses were performed with GraphPad Prism 7.0 (GraphPad Software, Inc. La Jolla, CA, USA). The experiments were performed in triplicates. Differences between groups were compared using one-way analysis of variance, and between two pairs data were analyzed by Student's t-tests. Continuous, normally distributed data were expressed as mean ± SD. Data followed by *p* < 0.05 were considered statistically significant.

### Ethics approval and consent to participate

The present study was approved by the Research Ethics Committee of The Guangxi Medical University Cancer Hospital.

## Results

### IKKα was up-regulated in residual TNBC tissues after chemotherapy and the adriamycin resistant TNBC cell line MDA-MB-231/ADR

**Figure 1:**
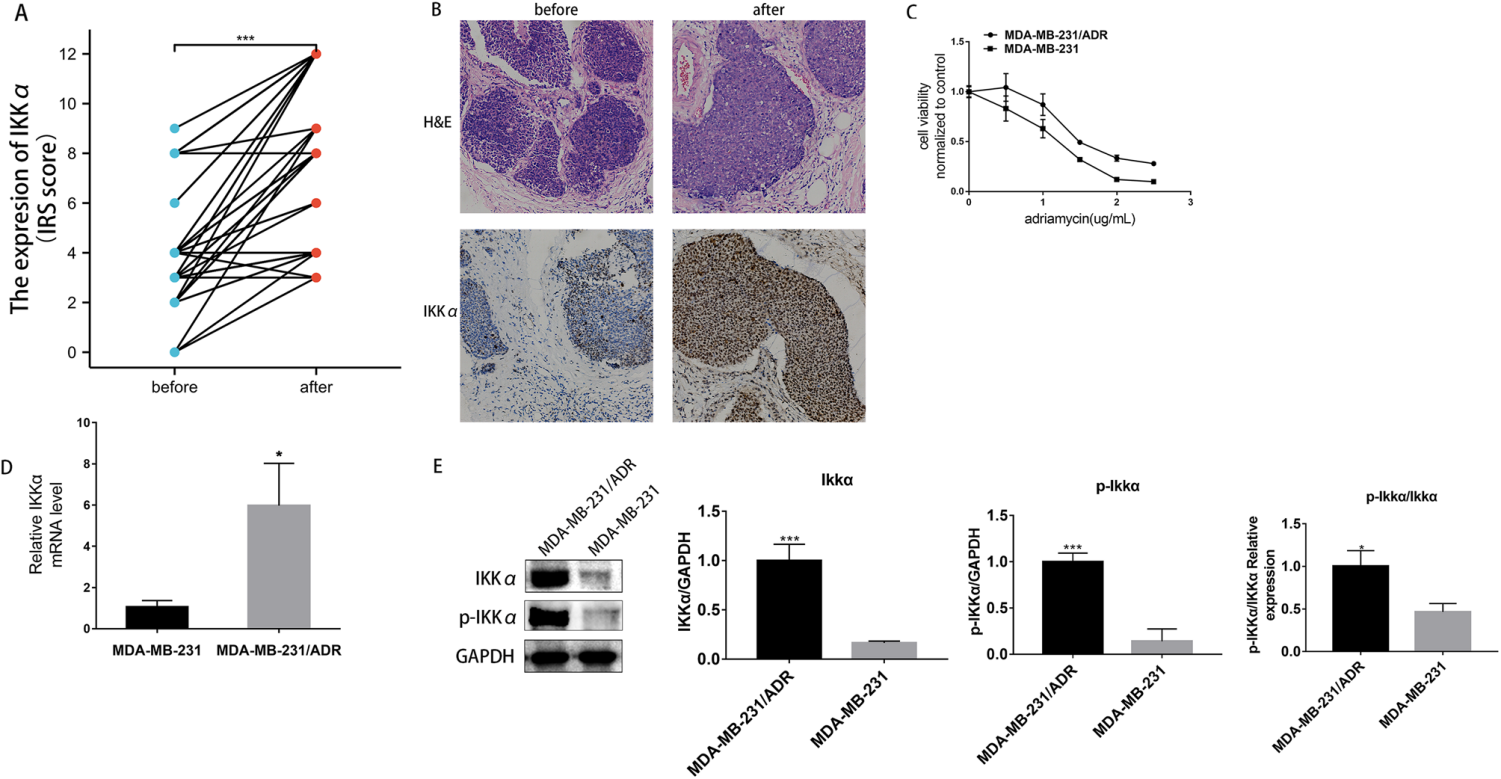
IKKα was up-regulated in residual TNBC tissues after chemotherapy and the adriamycin resistant TNBC cell line MDA-MB-231/ADR. (**A**) The expression of IKKα in TNBC tissues before and after neoadjuvant chemotherapy was detected by immunohistochemistry (Scale bars: 50 µm). (**B**) Representative images of H&E staining and IKKα expression by immunohistochemistry. (**C**) Detection of viability of MDA-MB-231 and MDA-MB-231/ADR cells after adriamycin treatment for 24 h by CCK-8 assay. (**D**, **E**) Detection of the mRNA and protein expressions of IKKα and phosphatase IKKα(p-IKKα) in MDA-MB-231 and MDA-MB-231/ADR cells by q-RTPCR and western blotting assay. *P < 0.05; ***P < 0.001.

A total of 43 surgical specimens were collected for analysis. Immunohistochemical results showed that IKKα expression was significantly high in the residual TNBC tissue after NAC than in the TNBC tumor tissue before NAC. Based on the defined IRS categories, 72.1% (31/43) of the non-pCR specimens were classifed as showing high expression of IKKα (IRS ≥ 6), while underexpression of IKKα level (IRS ≤ 4) was detected in 69.8% (30/43) of the cancers before neoadjuvant chemotherapy. To determine the relationship between IKKα and the pathologic response to neoadjuvant chemotherapy, all 43 breast cancer samples were stratified into 3 groups based on the Miller-Payne classification system: Grade-1/2, Grade-3, and Grade-4. The results showed that IKKα expression was higher in Grade-1/2 than in Grade-3 and Grade-4 tumors (*p* = 0.039), suggesting that high increased IKKα expression promotes or is associated with chemotherapy resistance (Fig. 1A,B).

To explore the relationship between the IKKα gene and adriamycin resistance in TNBC, we analyzed IKKα expression in adriamycin resistant in MDA-MB-231/ADR, a TNBC cell line that were generated by a pulse stimulated strategy. Drug sensitivities of MDA-MB-231 and MDA-MB-231/ADR cells to adriamycin were compared by CCK-8 assay. This analysis revealed that adriamycin significantly inhibited the viability of these two cell lines in a dose-independent manner (Fig. 1C). MDA-MB-231/ADR cells with IC50 ranging from 1.713 to 2.064 μg/mL were more resistant to adriamycin than MDA-MB-231 cells with IC50 ranging from 1.196 to 1.406 μg/mL. The q-RTPCR and western blot revealed that the expression of IKKα was higher in MDA-MB-231/ADR cells than in MDA-MB-231 cells (Fig. 1D,E).

### IKKα inhibition re-sensitizes acquired adriamycin-resistant TNBC cells to chemotherapy-induced apoptosis

**Figure 2:**
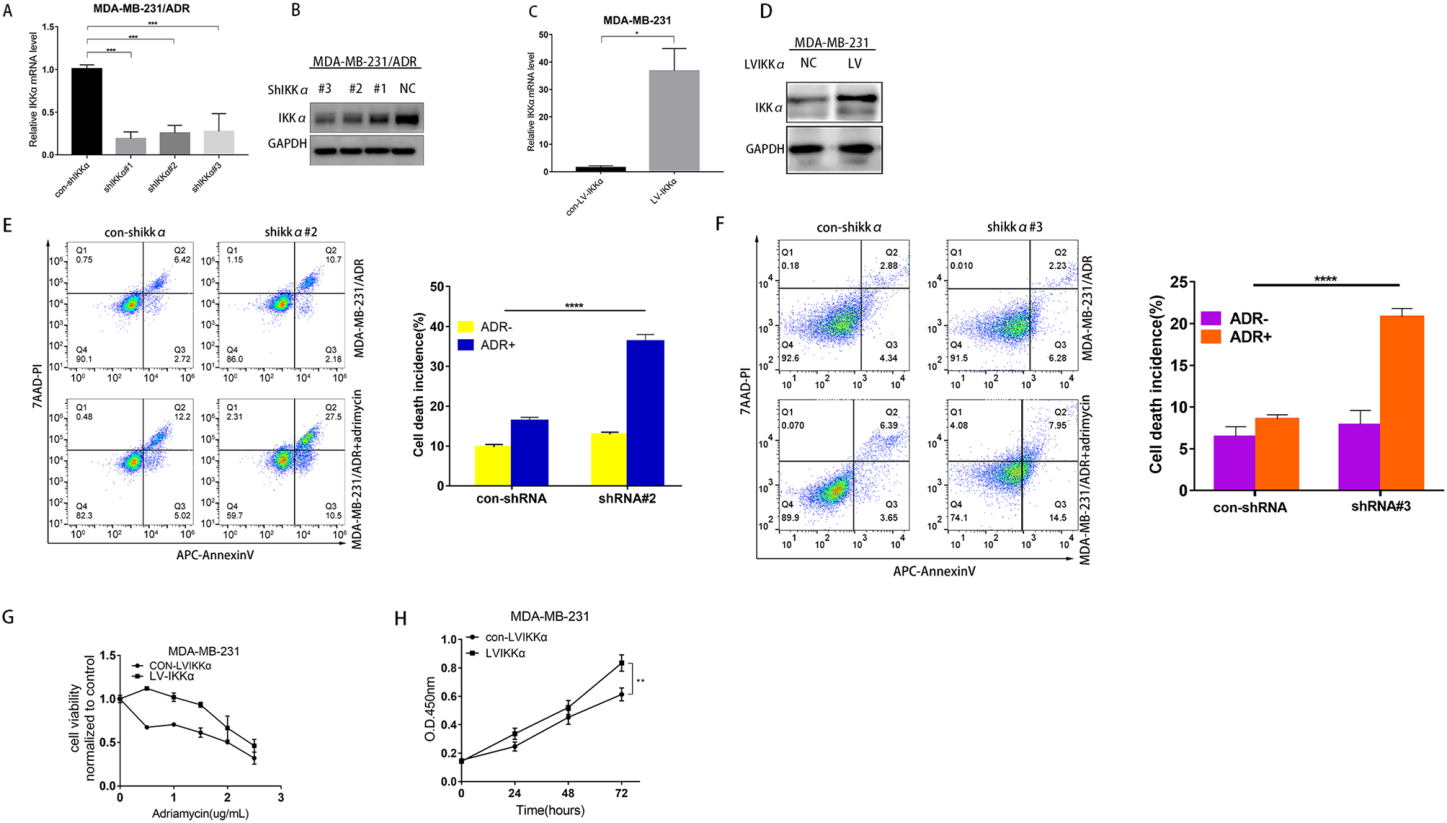
IKKα inhibition re-sensitizes acquired adriamycin-resistant TNBC cells to chemotherapy-induced apoptosis. (**A**) q-RTPCR analysis of IKKα overexpression efficiency in MDA-MB-231/ADR cells. (**B**) Western blotting analysis of IKKα knockout efficiency in MDA-MB-231/ADR cells. (**C**) q-RTPCR analysis of IKKα overexpression efficiency in MDA-MB-231 cells. (**D**) Western blotting analysis of IKKα overexpression efficiency in MDA-MB-231 cells. (**E**) The cell apoptosis incidence was detected by flow cytometric assay at 24 h after adrimycin exposure. Q1: necrosis; Q2: late apoptosis Q3: early apoptosis; Q4: live. (**F**) After IKKα downregulation, adriamycin induced cell apoptosis rate was significantly increased. (**G**) MDA-MB-231 cells were stably transfected with con-LVIKKαand LV-IKKα and then exposed to adriamycin of indicated doses for 24 h, cell viability was measured by CCK-8 assay. (**H**) The con-LVIKKα and LV-IKKα group were treated with dimethylsulfoxide (DMSO) or adriamycin (0.5 μg/mL) for 0 h, 24 h, 48 h, 72 h, cell viability was measured by CCK-8 assay. *P < 0.05; **P < 0.01; ***P < 0.001; ****P < 0.0001.

To investigate the role of IKKα in TNBC drug resistance, IKKα gene was knocked down and overexpressed by the lentivirus system. qPCR and western blot analyses confirmed IKKα gene knockdown efficiency. IKKα# 2 and IKKα#3 shRNA inhibited the transcription of mRNA and the expression of IKKα in MDA-MB-231/ADR cells most obviously (Fig. 2A,B). The overexpression efficiency of the IKKα gene is shown in Fig. 2C,D. LV-IKKαRNA overexpressed the mRNA transcription and the expression of IKKα protein in MDA-MB-231 cells (Fig. 2C,D) . Therefore, IKKα# 2 and IKKα#3 shRNA and LV-IKKαRNA were used in the subsequent experiments.

The role of IKKα in TNBC apoptosis was analyzed by flow cytometry. We found that 2 ug/mL adriamycin induced a higher apoptosis of MDA-MB-231/ADR cells in the IKKα#2 shRNA group than control IKKα shRNA group (38.0% vs. 17.22%);the IKKα#3 shRNA group than control IKKα shRNA group (21.95% vs. 10.04%) (Fig. 2E,F).

Next, we examined whether the overexpression of IKKα could induce adriamycin resistance in MDA-MB-231 cells. We found that the IC50 of MDA-MB-231 cells overexpressing IKKα (1.58–2.112 μg/mL) was higher than that of the control group (0.746–1.068 μg/mL) (Fig. 2G). In addition, after 72 h of adriamycin treatment, cell survival rates at different time points (0, 24, 36, and 72 h) showed that MDA-MB-231 cells overexpressing IKKα were more resistant to adriamycin than those underexpressing IKKα (Fig. 2H). Conversely, MDA-MB-231 cells with high IKKα expression were more likely to survive in adriamycin treatment, indicating that overexpression of IKKα promotes adriamycin resistance in TNBC.

### Knockdown of IKKα reduces the chemoresistance of MDA-MB-231 and MDA-MB-231/ADR cells against adriamycin and activates the mechanism of apoptosis

**Figure 3:**
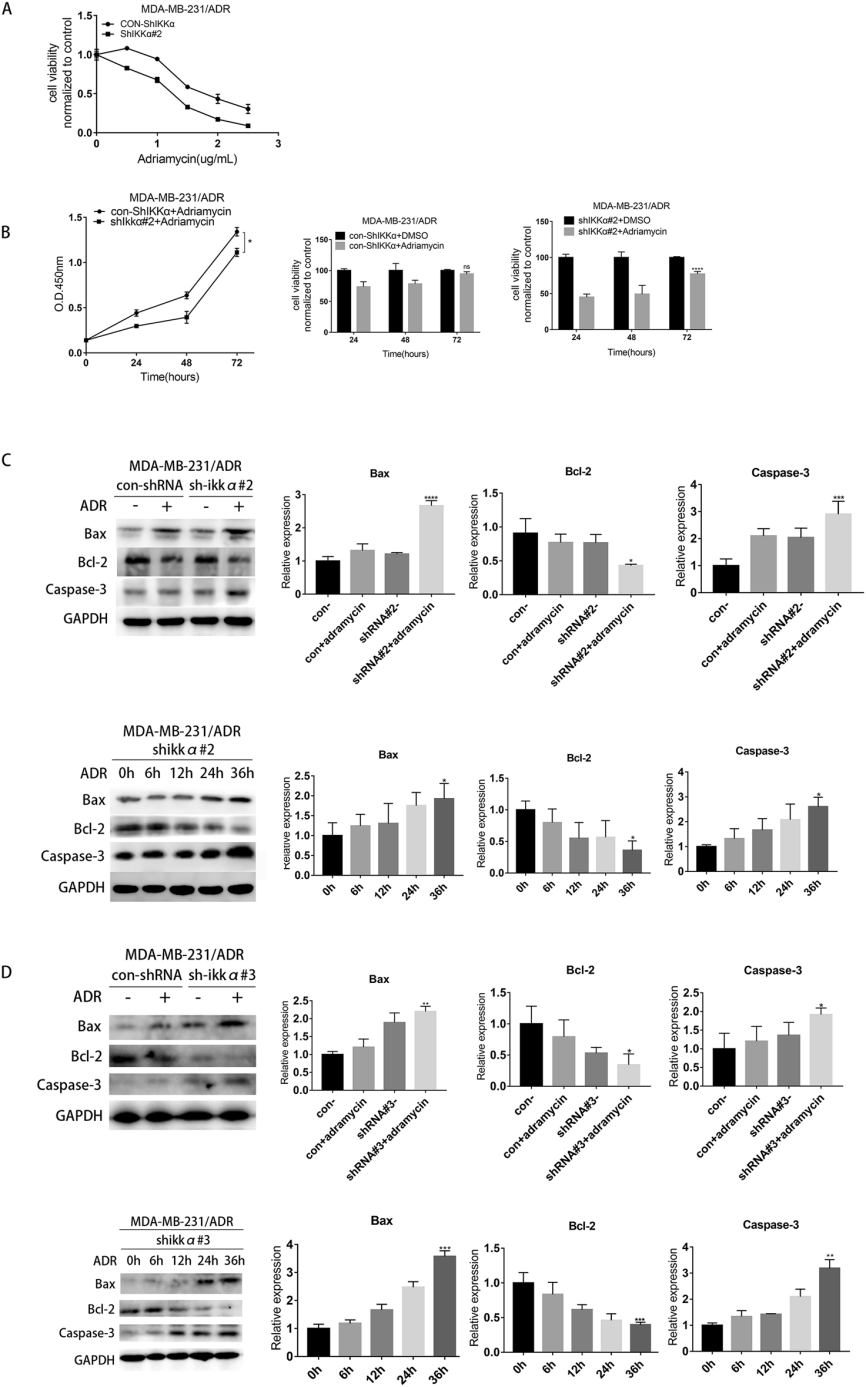
Knockdown of IKKα reduced the chemoresistance of MDA-MB-231/ADR cells to adriamycin. (**A**) MDA-MB-231/ADR cells were stably transfected with con-shIKKα and shIKKα#2 and then exposed to adriamycin of indicated doses for 36 h. Cell viabilitmly was measured by CCK-8 assay. (**B**) The con-shIKKα and shIKKα#2 group were treated with dimethylsulfoxide (DMSO) or adriamycin(0.5 μg/) for 0 h, 24 h, 48 h, 72 h. Cell viability was measured by CCK-8 assay. (**C**-**D**)Western blotting assay was used to evaluate the protein expression levels of apoptosis-related genes (Bax, Bcl-2 and cleaved caspase 3). *P < 0.05.

As previously described, IKKα expression in MDA-MB-231/ADR cells was markedly upregulated. Also, MDA-MB-231/ADR cells overexpressing IKKα were less sensitive to adriamycin than MDA-MB-231cells underexpressing IKKα. To further assess whether IKKα regulates the sensitivity of TNBC to adriamycin, we downregulated the expression of this protein in these cells. We found out that IC50 of MDA-MB-231/ADR cells underexpressing IKKα (1.125–1.244 μg/mL) was lower than that of the control-IKKα shRNA group (1.752–1.918 μg/mL) (Fig. 3A). Further analyses revealed that when cells were treated with the same concentration of adriamycin, MDA-MB-231/ADR cells underexpressing IKKα grow slower than the cells with high IKKα expression group (Fig. 3B). Meanwhile, compared with MDA-MB-231/ADR cells with high IKKα expression, the expression of pro-apoptosis proteins, including Bax and cleaved caspase 3 (c-caspase 3) were up-regulated in MDA-MB-231/ADR cells with IKKα down-regulated, and the expression of Bcl-2 was decreased. Changes in these apoptotic indicators were also positively correlated with exposure time to adriamycin in the underexpressing IKKα group (IKKα# 2 and IKKα# 3 shRNA group) (Fig. 3C,D). These results demonstrated that IKKα knockdown reduced the chemoresistance of TNBC cells to adriamycin.

### Inhibition of IKKα increases the sensitivity of adriamycin‑resistant breast cancer cells to adriamycin in vivo

**Figure 4:**
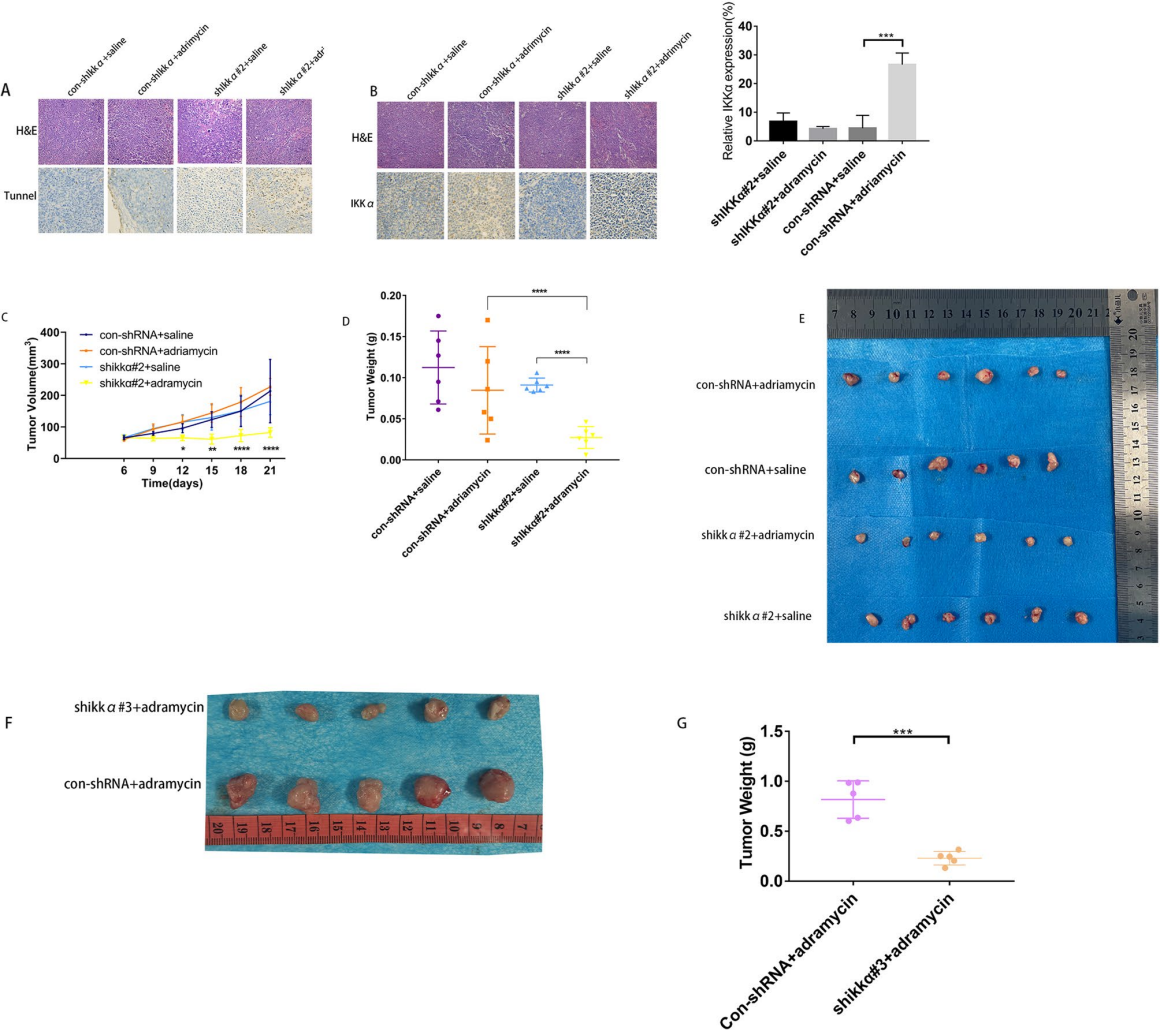
Inhibition of IKKα re-sensitizes adriamycin‑resistant breast cancer cells to adriamycin in vivo. (**A**) Tunnel assay showed that after IKKα downregulation, the cell apoptosis rate was significantly increased after adriamycin treatment. (**B**) The expression of IKKα protein in control-shIKKα + adriamycin group was higher than that of control-shIKKα + saline group. (**C**) The tumor-forming growth curves of nude mice treated with adrimycin or saline in the different groups. (**D**, and **G**) Tumours were harvested, and the tumour weights were measured in the different groups. (**E** and **F**) Tumors were removed from the nude mice and taken photograph to show the sizes of xenografted tumors. Statistical analysis of the weight of tumor excised from BALB/c nude mice at day 21. *P < 0.05; **P < 0.01; ***P < 0.001; ****P < 0.0001.

In order to confirm in vivo that inhibiting IKKα can restore the sensitivity of adriamycin resistant cells to adriamycin, we established transplanted tumor models of MDA-MB-231/ADR cells and MDA-MB-231/ADR cells with IKKα knockdown expression in mice, respectively, to observe the tumor inhibition after adriamycin treatment. The tunnel assay confirmed that in mice tumors formed by injection of underexpressing IKKα cells (shIKKα# 2 group) , the rate of apoptosis was higher in tumors treated with adriamycin than in tumors treated with saline(Fig. 4A). The results also showed that after treatment with adriamycin, the expression of IKKα protein was higher compared to that of tumor treatment with saline (Fig. 4B). Furthermore, In vivo experimental results showed that the tumor growth was slower in the IKKα low expression group, and the tumor volume and weight were significantly smaller than those in the normal saline treatment group and the IKKα high expression group(Fig. 4C,E–G), consistent with in vitro cell experiments (Supplementary file).

## Discussion

Chemotherapy is currently the mainstay adjuvant treatment of most TNBC^16^. Initially, TNBC was sensitive to cytotoxic chemotherapy, but a significant proportion has rapidly developed drug resistance^17^. The development of chemoresistance limits the efectiveness of chemotherap, and drug resistance is the main cause of cancer treatment failure^18^. Meanwhile, it is difficult to overcome acquired cancer resistance^19^. Therefore, identifying the molecular mechanisms contributing to breast cancer progression and chemoresistance could provide novel biomarkers for the precise prediction of patient prognosis and for molecular targeted therapy. Studies have shown that evasion of apoptosis is integral to drug resistance^20–24^. For instance, our previous studies have demonstrated that IKKα is highly expressed in tumor cells during arsenic-induced chemotoxic injury and is associated with apoptosis^11^. In this study, we found that the expression of IKKα in MDA-MB-231/ADR cells and in drug-resistant residual TNBC tumor tissues after neoadjuvant chemotherapy was significantly up-regulated, providing evidence that IKKα plays a key role in acquired drug resistance of triple-negative breast cancer.

Moreover, IKKα knockdown induced intrinsic apoptosis in MDA-MB-231/ADR cells. Further experiments showed that downregulating the expression of IKKα increased the sensitivity of MDA-MB-231/ADR cells to adriamycin in vitro and inhibited the growth of MDA-MB-231/ADR cells in vivo. Overexpression of IKKα increased the chemoresistance of MDA-MB-231 cells to adriamycin in vitro. Therefore we concluded that IKKα exerts an important and previously unknown role in promoting chemoresistance in TNBC, combining IKKα inhibition with chemotherapy may be an effective strategy to improve treatment outcome in chemoresistant TNBC patients.

At present, there are few studies on IKKα and chemotherapy resistance of tumors, and the correlation between IKKα and chemotherapy resistance of breast cancer is more limited. The present study focused on the role of IKKα in adriamycin resistance of TNBC. It is well-accepted that constitutive NF-κB signaling activation promotes cancer development by increasing cell proliferation and resistance to apoptotic stimuli^25^, the IKK kinase complex (IKKα and IKKβ) is the master regulator for NF-κB activation^26^. However, the function of the IKK kinase complex is not limited to the well-known NF-κB signaling pathway. Previously, we demonstrated the NF-κB-unrelated cytoprotective function of IKKα in promoting autophagy in the arsenite-treated hepatoma cells^11^. Colomer C et al. also demonstrated that IKKα kinase regulates the DNA damage response and drives chemotherapy resistance in cancer, independent of NF-κB activation^7^. The role of IKKα in acquired drug resistance of breast cancer induced by chemotherapeutic drugs need to be further investigated. Here, we identified a positive correlation between IKKα overexpression and TNBC drug resistance. A subpopulation of chemotherapy-resistant residual tumor cells remaining in breast tissue could be responsible for the high metastatic recurrence rates and poor long-term clinical outcomes of TNBC^27^. IKKα expression was high in more than 70% of the residual tumors, while underexpression of IKKα level was detected in 81.4% of the cancers before neoadjuvant chemotherapy. High expression of IKKα positively correlated to lower MP grades. Tumor disappearance was lowest, while residual tumour tissue was highest after neoadjuvant chemotherapy in the MP 1 to 2 groups. The residual tumor tissues were highly resistant to chemotherapy. Therefore, the expression levels of IKKα in residual tumor tissue of MP1-2 group were higher than those in the other groups, suggesting that high IKKα levels are associated with chemotherapy resistance of TNBC. In general, both in vitro and in vivo experiments revealed that underexpression of IKKα reduced the chemoresistance of MDA-MB-231/ADR cells to adriamycin, and overexpression of IKKα induced adriamycin resistance in MDA-MB-231 cells. In human fibrosarcoma cells, Bednarski BK et al.^28^ have shown that IKKα plays a key role in doxorubicin resistance and may serve as a potential target for combination strategies to improve chemotherapy response, consistent with our current findings. Colomer et al.^7^ demonstrated that IKKα inhibition synergistically enhance the therapeutic potential of the standard of care therapy in colorectal cancer (5-FU plus irinotecan), leading to the eradication of chemotherapy-resistant metastatic human tumors in vivo. Our results, together with these findings, provide a rationale for the use of IKKα inhibitors as a novel combination strategy for cancer treatment, even for patients who have developed resistance to single chemotherapy regimen.

In the present study, the mechanism underlying IKKα-dependent adriamycin resistance was further uncovered. Our previous study has shown that IKKα is critical for mediating the pro-apoptotic effect of arsenite, a cytotoxic chemical reagent. Thus, we detected the expression of the pro-apoptotic protein Bax and the antiapoptotic protein Bcl-2 in IKKα-mediated adriamycin resistant TNBC cells. Our findings suggest that compared with MDA-MB-231/ADR cells, the expression of Bax was up-regulated in MDA-MB-231/ADR cells with IKKα down-regulated, and the expression of Bcl-2 was decreased. Activation of caspase-3 expression is the final step of the apoptosis cascade^29^, our study also showed that downregulating IKKα expression combined with adriamycin therapy significantly enhanced the expression of cleaved caspase 3. Dysregulation of the apoptotic cell death machinery is a hallmark of cancer. Altered apoptosis is implicated not only related to the development and progression of tumor, but also related to the resistance of tumor to therapy. Most of the anticancer drugs currently used in chemotherapy trigger cancer cell death by activating the apoptosis signaling pathway. Thus, the protective effect of apoptosis may lead to drug resistance, which limits the effectiveness of treatment^30^. The p53 signaling pathway is a recognized regulatory pathway related to cell apoptosis^2,31^. which can regulate the expression of apoptosis-related genes, such as Bax and Bcl-2, members of the Bcl-2 family^32^. The balance and protein–protein interactions between Bcl-2 family members is required to determine whether a cell undergoes cell survival or apoptosis. Adriamycin resistance in breast cancer cells has been shown to be associated with downregulation of Bcl‐2 expression and up-regulation of Bax expression^33,34^. Consistent with this, our results suggested that IKKα's regulation of apoptotic protein expression is an important factor leading to adriamycin resistance in MDA-MB-231 cells, and down-regulation of IKKα expression can enhance the effect of adriamycin on MDA-MB-231/ADR TNBC cells by inhibiting Bcl-2/Bax signaling pathway.

In summary, our results indicated that IKKα was up-regulated in adriamycin resistant TNBC cells and residual tissues after chemotherapy, and its expression was associated with the response to neoadjuvant chemotherapy. In addition, IKKα inhibition re-sensitizes acquired adriamycin-resistant TNBC cells to chemotherapy-induced apoptosis. These findings suggest that IKKα exerts an important and previously unknown role in promoting chemoresistance in TNBC, combining IKKα inhibition with chemotherapy may be an effective strategy to improve treatment outcome in chemoresistant TNBC patients.

## Supplementary Information

Supplementary Information.

## Data Availability

The datasets used and/or analyzed during the present study are available from the corresponding author on reasonable request.
