# Supplementary material for: IKKα inhibition re-sensitizes acquired adriamycin-resistant triple negative breast cancer cells to chemotherapy-induced apoptosis
Source: Sci Rep. 2023 Apr 17;13:6211. doi: 10.1038/s41598-023-33358-x (PMC10110611; doi:10.1038/s41598-023-33358-x)
Supplement: Supplementary file 1 — Supplementary Information. [file 41598_2023_33358_MOESM1_ESM.pdf]

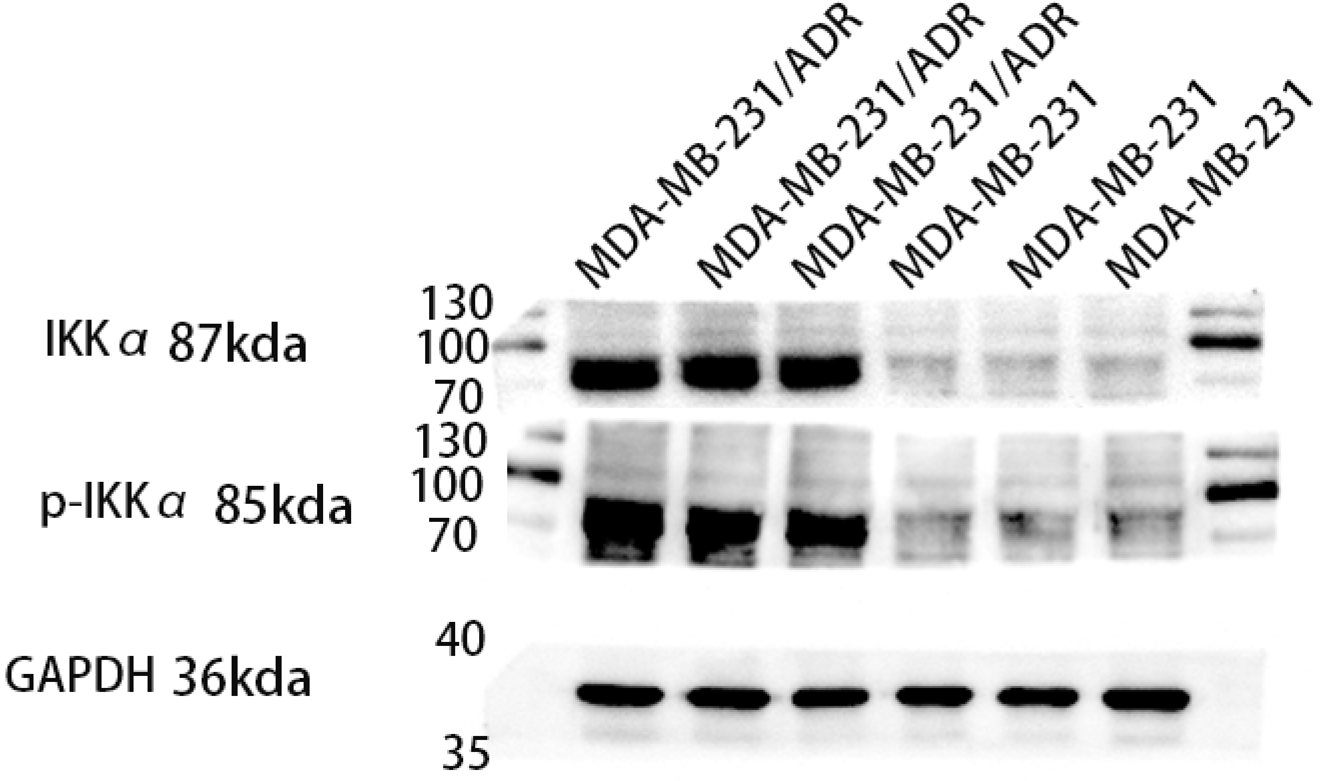

Apply to FIGURE1 E
IKKα and p-IKKα express

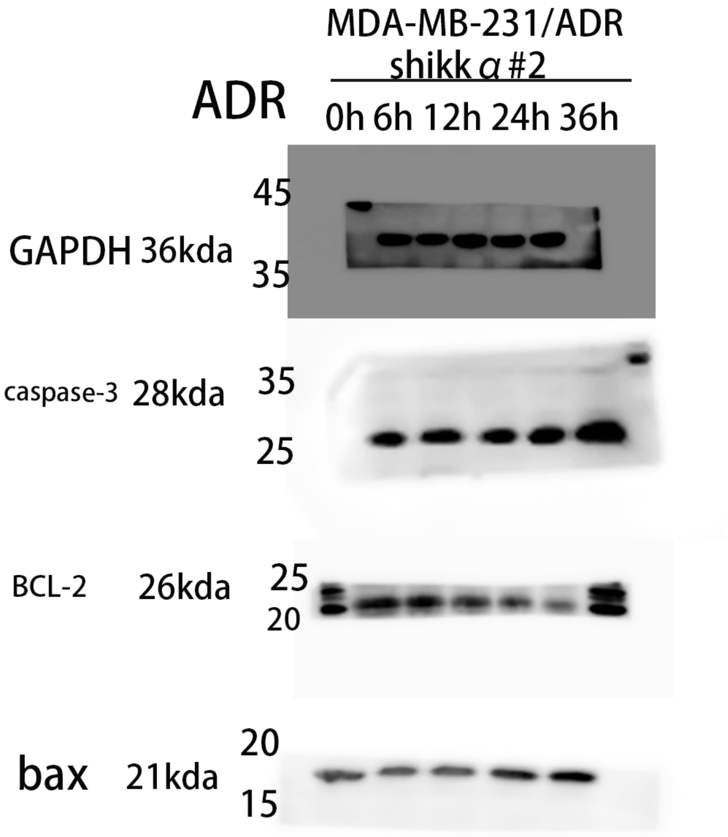

Apply to FIGURE3 C Changes of apoptotic proteins over time after IKKα knockdown

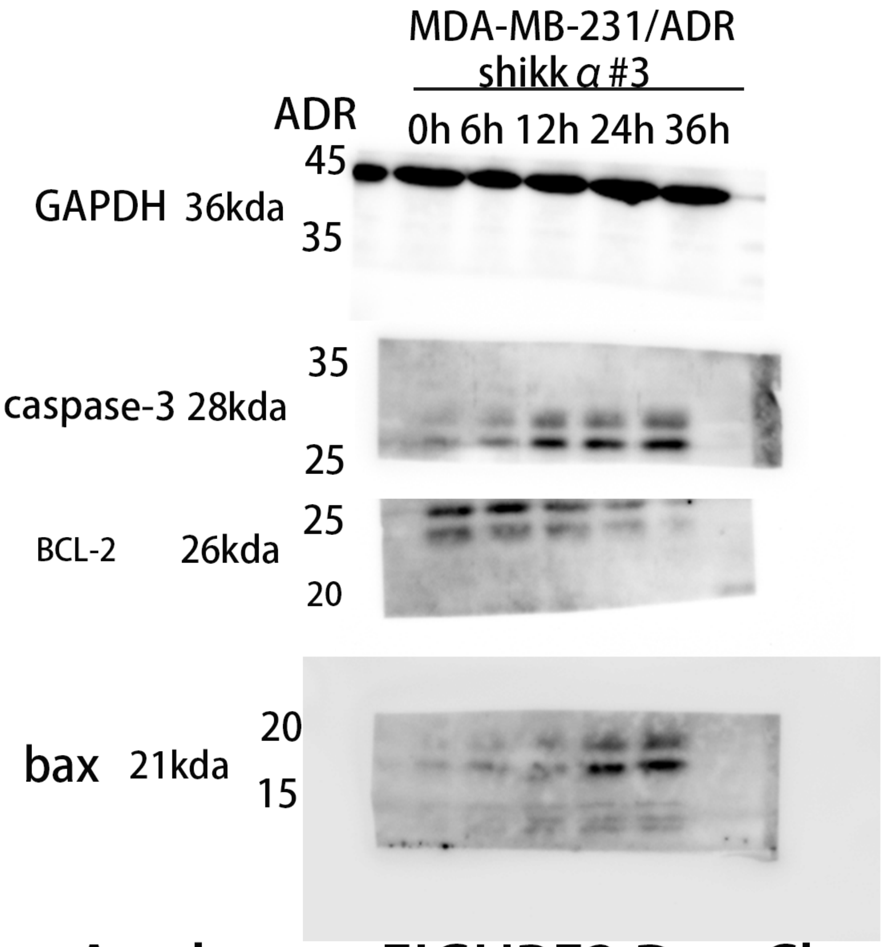

Apply to FIGURE3 D Changes of apoptotic proteins over time after IKKα knockdown

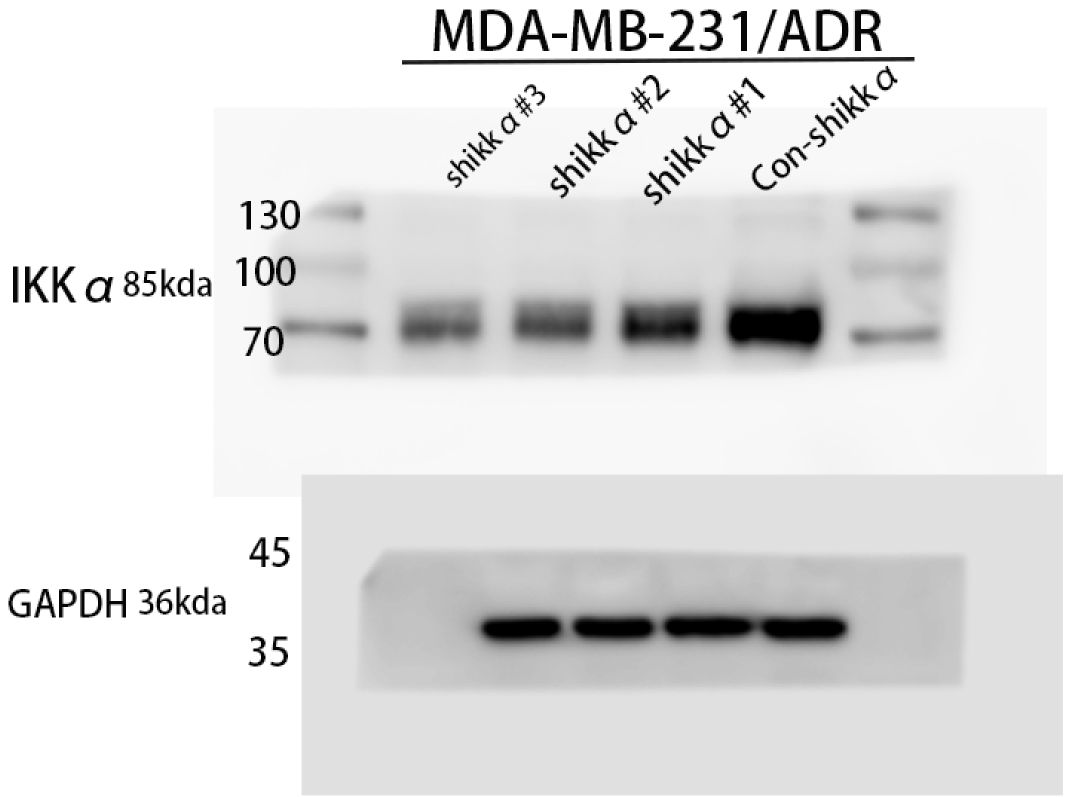

Apply to FIGURE2 B Knockdown IKKα

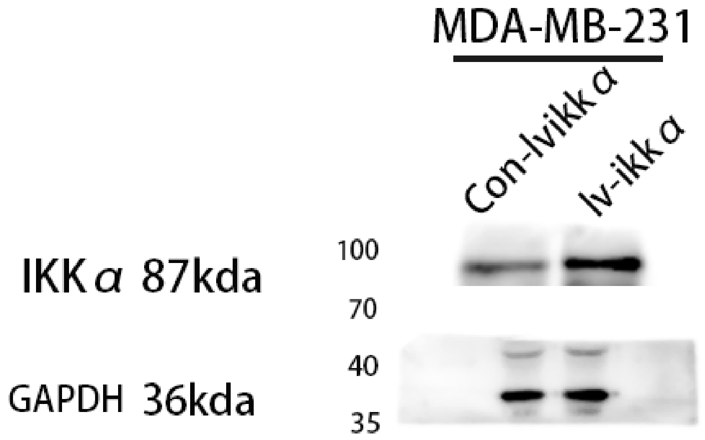

Apply to FIGURE2 D overexpress IKKα

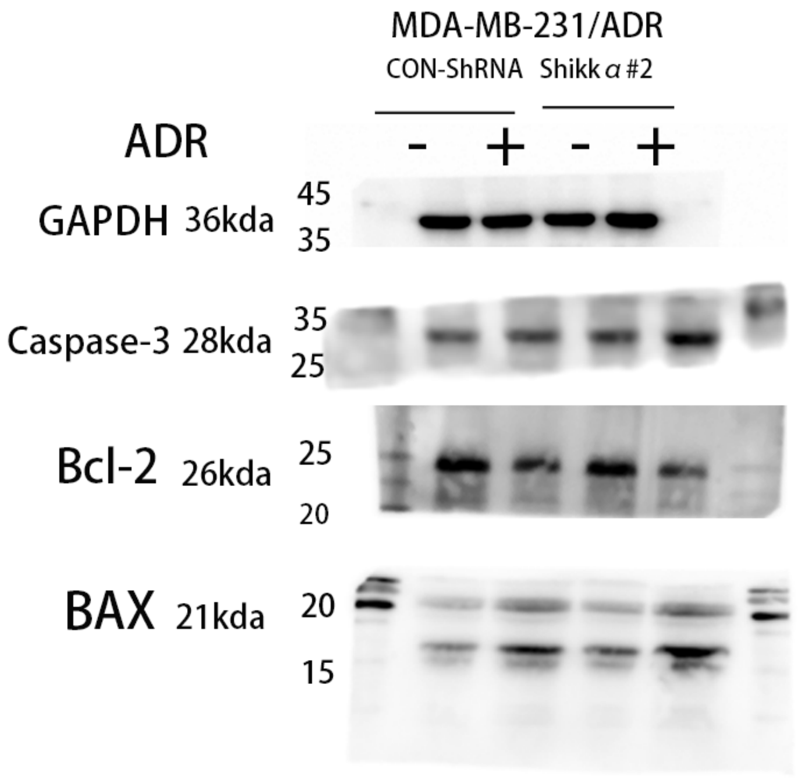

Apply to FIGURE3 C Changes of apoptotic proteins after IKKα knockdown

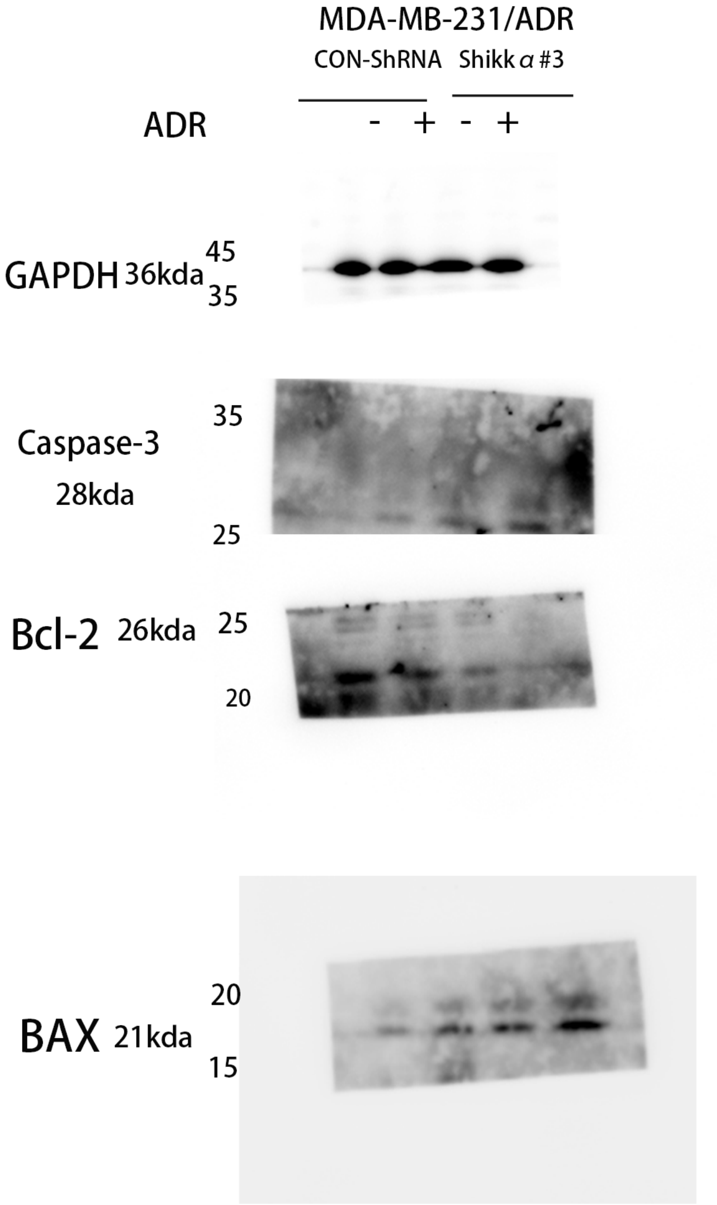

Apply to FIGURE3 D Changes of apoptotic proteins after IKKα knockdown
